# Deep learning enables pathologist-like scoring of NASH models

**DOI:** 10.1038/s41598-019-54904-6

**Published:** 2019-12-05

**Authors:** Fabian Heinemann, Gerald Birk, Birgit Stierstorfer

**Affiliations:** Drug Discovery Sciences, Boehringer Ingelheim Pharma GmbH & Co. KG, 88397 Biberach an der Riß, Germany

**Keywords:** Machine learning, Drug development, Metabolic syndrome, Drug discovery

## Abstract

Non-alcoholic fatty liver disease (NAFLD) and the progressive form of non-alcoholic steatohepatitis (NASH) are diseases of major importance with a high unmet medical need. Efficacy studies on novel compounds to treat NAFLD/NASH using disease models are frequently evaluated using established histological feature scores on ballooning, inflammation, steatosis and fibrosis. These features are assessed by a trained pathologist using microscopy and assigned discrete scores. We demonstrate how to automate these scores with convolutional neural networks (CNNs). Whole slide images of stained liver sections are analyzed using two different scales with four CNNs, each specialized for one of four histopathological features. A continuous value is obtained to quantify the extent of each feature, which can be used directly to provide a high resolution readout. In addition, the continuous values can be mapped to obtain the established discrete pathologist-like scores. The automated deep learning-based scores show good agreement with the trainer - a human pathologist.

## Introduction

Non-alcoholic fatty liver disease (NAFLD) and non-alcoholic steatohepatitis (NASH) are among the most relevant diseases in terms of prevalence^1^, reduction of quality of life^2^, increase in mortality^3^ and socio-economic burden on a global scale^4^.

NAFLD is characterized by an excess of fat (steatosis) in the liver. About 25% of the global adult population is affected and the prevalence is increasing^1^. Patients with NAFLD often exhibit obesity, diabetes, hypertension and an increased waist circumference, which are features of metabolic syndrome^1^. NAFLD can progress to NASH, a disease where the liver of the patient is additionally affected by varying degrees of cell death, inflammation, and collagen deposition. Prevalence estimates for NASH range from 1.5–6.5%^1^. Patients with NASH can develop cirrhosis^5^, liver failure^6^, and possibly hepatocellular carcinomas^7^.

For the treatment of NAFLD, weight loss and lifestyle changes (e.g. exercise and change in diet) are recommended interventions^8,9^. It is also beneficial to have supporting therapeutic options at hand which directly modulate relevant molecular pathways of the liver. However, to date, no approved therapies for NASH/NAFLD are available^9–11^, though several novel compounds are in clinical trials^12^.

To study the efficacy of novel compounds from preclinical research, animal models of liver disease are required until substitute systems can fully mimic the complexity of the liver within an organism (e.g. ‘organoids’ or ‘organs on a chip’^13,14^). Frequently used models are based on the rat or mouse, where a pathophysiology comparable to NAFLD/NASH is induced by a high-fat diet or substances like CCl_4_^15–17^.

A typical pre-clinical efficacy study consists of a healthy control group, a disease model group, and one or more disease model groups with additional administration of a novel compound. Frequently used readouts of efficacy studies in NAFLD/NASH are biochemical parameters, quantitative image analysis and pathologist scoring of histopathological sections.

A widely used pathologist score is based on Kleiner and co-workers (in the following also referred to as the ‘Kleiner score’), who demonstrated that ballooning, inflammation, steatosis, and fibrosis are the main histopathological features that correlate with the diagnosis of NASH^18^. The first three features are considered reversible. They are quantified in discrete so-called sub-scores (ballooning: 0–2, inflammation: 0–3, steatosis: 0–3) and summed up in the ‘NAS score’ (NAFLD activity score, 0–8). In addition, the mostly non-reversible fibrosis is quantified separately using a discrete fibrosis score of 0–4. These definitions of the respective discrete values for the four histopathological features rely on morphological structures (e.g. the presence of no, few, or many ballooning cells per liver for ballooning sub-scores 0, 1, or 2) which can be assigned by a trained pathologist by microscopic investigation. Typically this is done using hematoxylin and eosin (H&E) or Masson’s trichrome stained slides. Whereas human-based scoring is worldwide accepted, it has its drawbacks. First, it relies on expert pathologists, an in-demand occupation^19^. The task is time-consuming and can be tiring, which may consequently affect performance. Second, it was shown that produced results exhibit inherent variability between different pathologists^18^ and the same pathologist^20^. This can limit the comparability of the results. Third, the score based on Kleiner and colleagues produces discrete readouts with only a few unique steps which can be too coarse to quantify the effects of a compound.

Recent advances in deep learning^21^, and in particular in convolutional neural networks (CNNs, a type of deep learning used in image recognition), have revolutionized image analysis^22^ and are assumed to show at least human-like performance in image classification^23^. Due to these developments, complex image recognition tasks which were previously the exclusive domain of humans are now on the verge of automation. The number of applications in histopathology is rapidly growing. Examples include prostate cancer diagnosis^24^, non-small cell lung cancer diagnosis and mutation prediction^11^ or pathologist-like fibrosis and inflammation scoring in lung tissue^25^.

Here we describe a simple deep learning-based approach to automate the Kleiner score for NAFLD/NASH models in the rat and mouse. For each histopathological feature (fibrosis, ballooning, inflammation, steatosis), continuous quantities are generated which can be used directly as high-resolution readouts. Furthermore, we show an approach for mapping these continuous quantities to discrete pathologist scores. With corresponding training data, the method can be easily transferred to human tissue to build diagnostic systems.

## Results and Discussion

**Figure 1:**
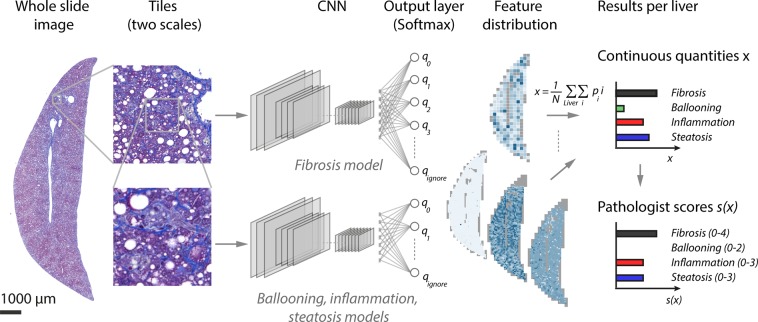
Overview of the workflow for the automated scoring of fibrosis, ballooning inflammation and steatosis, the features correlated with non-alcoholic fatty liver disease (NAFLD)/non-alcoholic steatohepatitis (NASH). A whole slide scan stained with Masson’s trichrome is cut into tiles of two scales. Low magnification tiles are analyzed by a convolutional neural network (CNN) to recognize fibrosis. High magnification tiles are analyzed by three separated CNNs to recognize ballooning, inflammation and steatosis. The probability-like output of the CNNs per tile results in the distribution of the histopathological features in the liver. The distributions are aggregated to obtain one continuous quantity per liver sample for each of the features. The continuous features can be used directly or mapped to discrete pathologist-like scores.

Figure 1 shows an overview of the automated Kleiner score for NAFLD/NASH. Our workflow is based on tissue sections stained with Masson’s trichrome, a stain highlighting collagen (stained in blue/violet) in contrast to other tissue structures (stained in red/purple). After microscopy using a whole slide scanner, the images were cut into image tiles of two dimensions: low magnification tiles (1.32 µm/px) to identify fibrosis and high magnification tiles (0.44 µm/px) to identify ballooning, inflammation and steatosis. Subsequently four different CNNs were used to classify the tiles according to the four histological features. The spatially resolved results were then aggregated to obtain a single continuous readout per sample. In addition, the continuous readout was mapped to Kleiner’s discrete pathologist-like sub-scores on ballooning (0–2), inflammation (0–3), steatosis (0–3), and the fibrosis score (0–4).

A prerequisite for the classification were trained CNNs that could recognize the relevant features. For this purpose, a trained pathologist with >5 years of experience in scoring liver sections created training data for each of the four histopathological features. Classes were defined in such a way that they were simple to assign and to allow a mapping to the Kleiner score.

**Figure 2:**
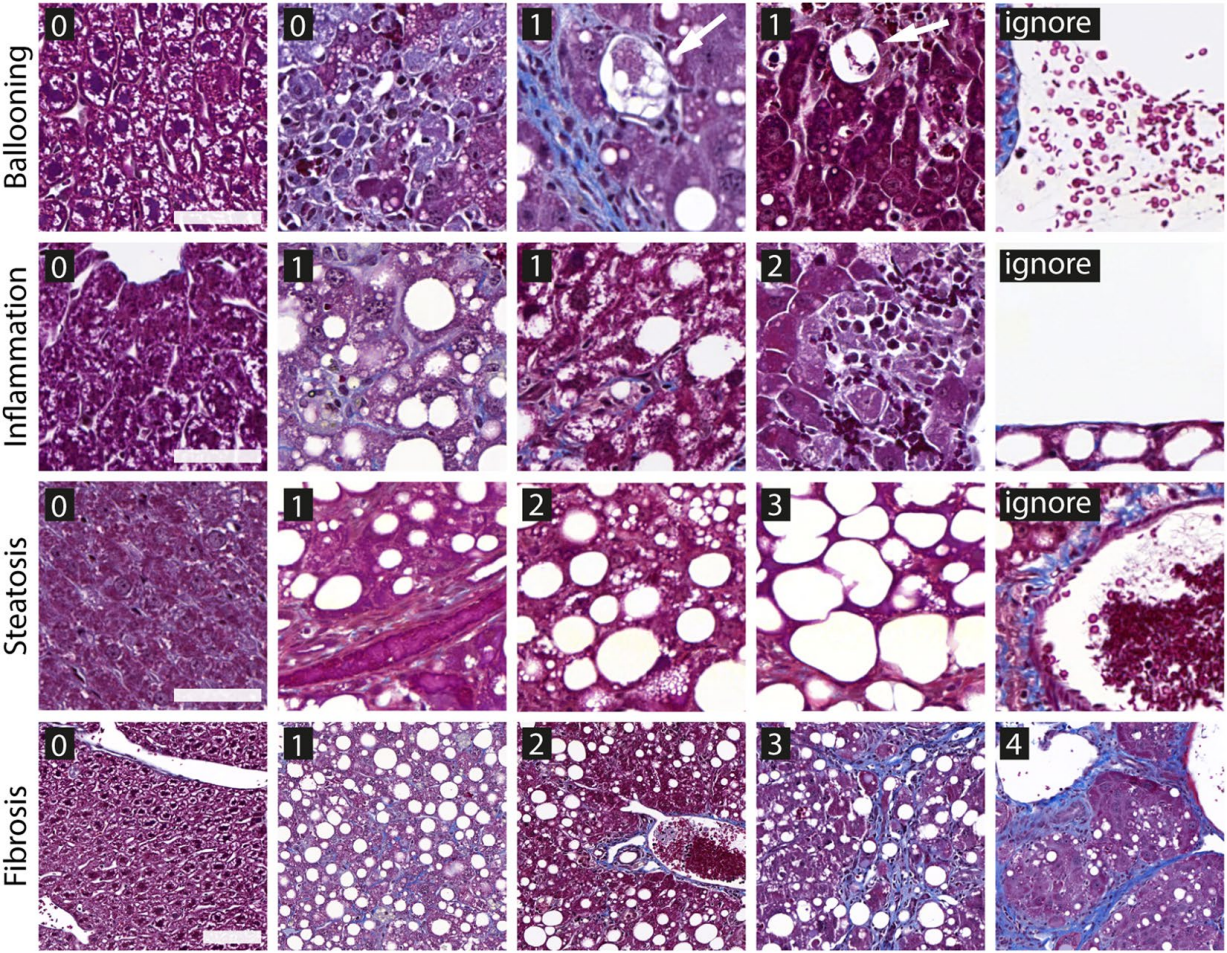
Examples of the classes used to train the four convolutional neural networks (CNN) to recognize relevant features of the histopathological features in the Kleiner score. First row: ballooning, with classes 0 (no-ballooning), 1 (ballooning cells, see arrows) and ignore (insufficient liver tissue visible on a tile). Second row: inflammation with classes 0 (no inflammation), 1 (moderate inflammation), 2 (severe inflammation with clear inflammatory foci) and ignore. Third row: steatosis with classes 0 (<5% area coverage of vacuoles), 1 (>=5% and <33%), 2 (>=33% and <66%), 3 (>=66%) and ignore. Fourth row: fibrosis with classes 0, 1 (perisinosiodal or periportal fibrosis), 2 (perisinosiodal and periportal fibrosis), 3 (bridging fibrosis), 4 (cirrhosis) and ignore (not shown). Scale bars in first three rows (high magnification tiles): 50 µm, last row (low magnification tiles): 100 µm.

Figure 2 shows examples of the classes used to identify the four histological features. The classes for ballooning are shown in the top row. In ballooning the cytoplasm clears and the cells are larger as the neighboring hepatocytes (see arrows in the top row of Fig. 2). A simple class definition of 0 (no ballooning cell on a tile) and 1 (a definite ballooning cell on a tile) was used. The classes for inflammation are shown in the second row. Tile classes of 0 (no inflammation), 1 (moderate inflammation) and 2 (severe inflammation with clear inflammatory foci; see materials and methods for the exact definition) were used. Here the distinction between 1 and 2 was required to allow the discrimination between different degrees of inflammation on a macroscopic scale (per liver). A hypothetical scoring system with only 0 and 1 would not allow differentiation between cases with weak inflammation in all tiles and cases with severe inflammation in all tiles and therefore have no resolution for higher values of the inflammation sub-score. Classes for steatosis are shown in the third row. Here the tile classes 0, 1, 2, and 3 directly correspond to the macroscopic steatosis sub-score since the macroscopic sub-score is based on area covered by vacuoles, which is directly applicable to the tile level. Finally, classes for fibrosis are shown in the fourth row. In this case the low magnification tiles were used (three times the length compared to small tiles) since structures such as bridging fibrosis (class 3) and cirrhosis (class 4) require a larger field of view per tile to reveal sufficient information for identification. Also in this case, the classes of the tiles corresponded to the macroscopic definition of fibrosis since the features were already recognized on the tile level (but may vary over the tissue section). In all four models, we used an “ignore” class to identify all cases where liver tissue was not-sufficiently visible on a tile or other artifact types were present (e.g. out of focus, mostly blood, or staining artifacts), as in our previous work^25^. The “ignore” class ensured, that only tiles containing actual liver tissue were further analyzed.

Subsequently the four CNN models were trained on ~90% of the annotated tiles and evaluated using the remaining tiles (validation set). Table 1 presents the per tile classification performance after model training. In all cases, the CNNs resulted in high classification accuracies of 86.0–94.5% on the unseen validation data.

**Table 1:** Accuracy of tile classification performance of the four convolutional neural network (CNN) models on ballooning, inflammation, steatosis and fibrosis.

	Classification accuracy		N		Number of classes including “ignore”
	Train	Validation	Train	Validation	
Ballooning	94.0%	93.1%	13590	1555	3
Inflammation	86.2%	86.0%	8567	914	4
Steatosis	93.8%	94.5%	6377	737	5
Fibrosis	88.5%	86.3%	4251	465	6

N is the number of labeled tiles used for training and validation.

**Figure 3:**
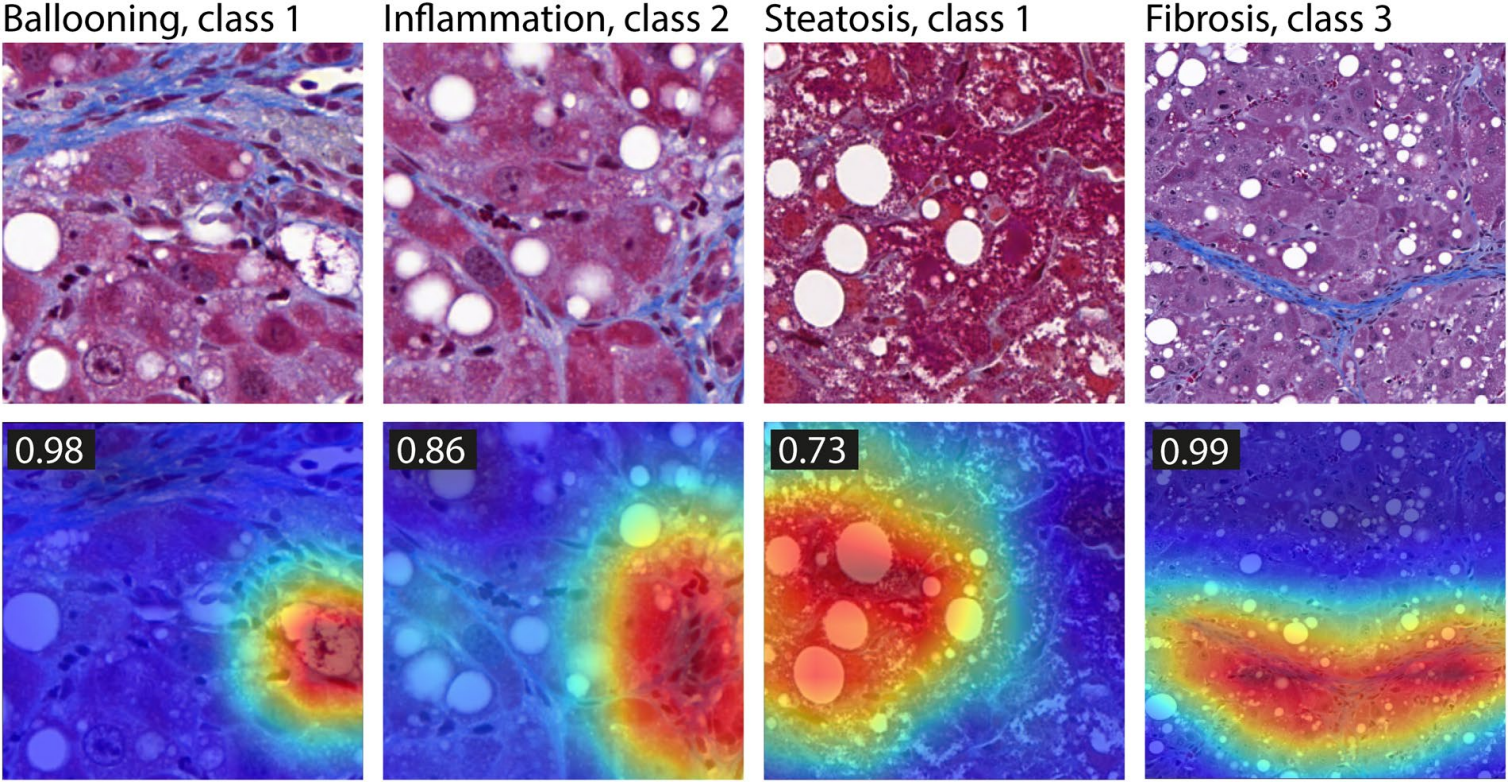
Examples of class activation maps for the four convolutional neural network (CNN) models. The color-coded maps highlight regions which were most discriminative for a certain class (for example a ballooning cell or a fibrotic band [CNN model and the selected class are shown on top]). Numbers on the top left in the activation map images are output probabilities for the respective classes.

To visualize that the CNN models in fact learned the relevant histological features, we applied class activation maps, a method to identify image regions which were the most relevant for a classification^26^. Figure 3 presents examples for all four models, indicating that the models learned the morphologically relevant features (i.e. ballooning cells, inflammatory foci, steatotic areas and fibrotic bands). Methods for visual feature explanation are important for excluding irrelevant structures in the training data being used for classification, a potential issue that can be caused by non-representative training data^27^.

We further computed confusion matrices to quantitatively analyze whether certain types of images are particularly difficult to classify (Fig. S1). Most of the cases are either exactly right (values on diagonal) or the classified class deviates by only +/−1 compared to the ground truth, i.e. with a neighboring class. This is mostly due to two factors: First, some cases will represent transitions between the discrete classes (for example between fibrosis class 0 and 1), and second, some cases are inherently difficult to classify. For example, in the ballooning CNN model, the presence of simultaneous cell debris and steatosis could, in a number of cases, resemble ballooning; using the example of inflammation, in a few cases it was challenging to discriminate red blood cells from inflammatory cells, i.e. when the staining was very dark. These cases were generally challenging for both the pathologist and the CNN models. However, for the predominant majority of cases the high levels of tile recognition accuracy and the confusion matrix analysis show that the CNN model classifications are in very good agreement with the pathologist.

**Figure 4:**
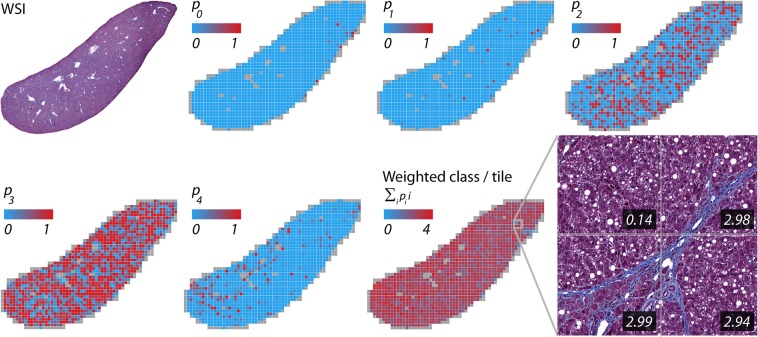
Conversion of individual class scores to the weighted class per tile using the fibrosis model as an example. A whole slide image (WSI) of a liver section stained with Masson’s trichrome (top left) and the corresponding distribution of the normalized neural network output confidences for the fibrosis classes 0, 1, 2, 3 and 4. In this example, the majority of tiles are classified as fibrosis class 3. The output confidences are summarized as weighted class per tile. The insets show four tiles of a region with neighboring bridging fibrosis and an almost unaffected area. Numbers correspond to the weighted class per tile.

After training of the four classification CNNs, these models were applied to new images. As a result, a distribution of probability scores for the respective classes was obtained for the whole tissue sample. Figure 4 shows an example for the fibrosis model applied to a case of a fibrotic liver. Clearly, the majority of cases distribute around fibrosis class 3 (“bridging fibrosis”). The individual output probabilities are summarized to a single value, the weighted class per tile. The inset shows examples of a region with bridging fibrosis and an example of no fibrosis next to each other.

For the transition from individual tiles to the macroscopic scores per liver sample, the average of all tiles was computed, i.e. the average weighted class per liver section. This continuous readout has a range equivalent to the number of classes in each model, i.e. ballooning 0–1, inflammation 0–2, steatosis 0–3, and fibrosis 0–4. In the future, these continuous readouts might be used as improved readouts describing the extent of the four histological features.

**Figure 5:**
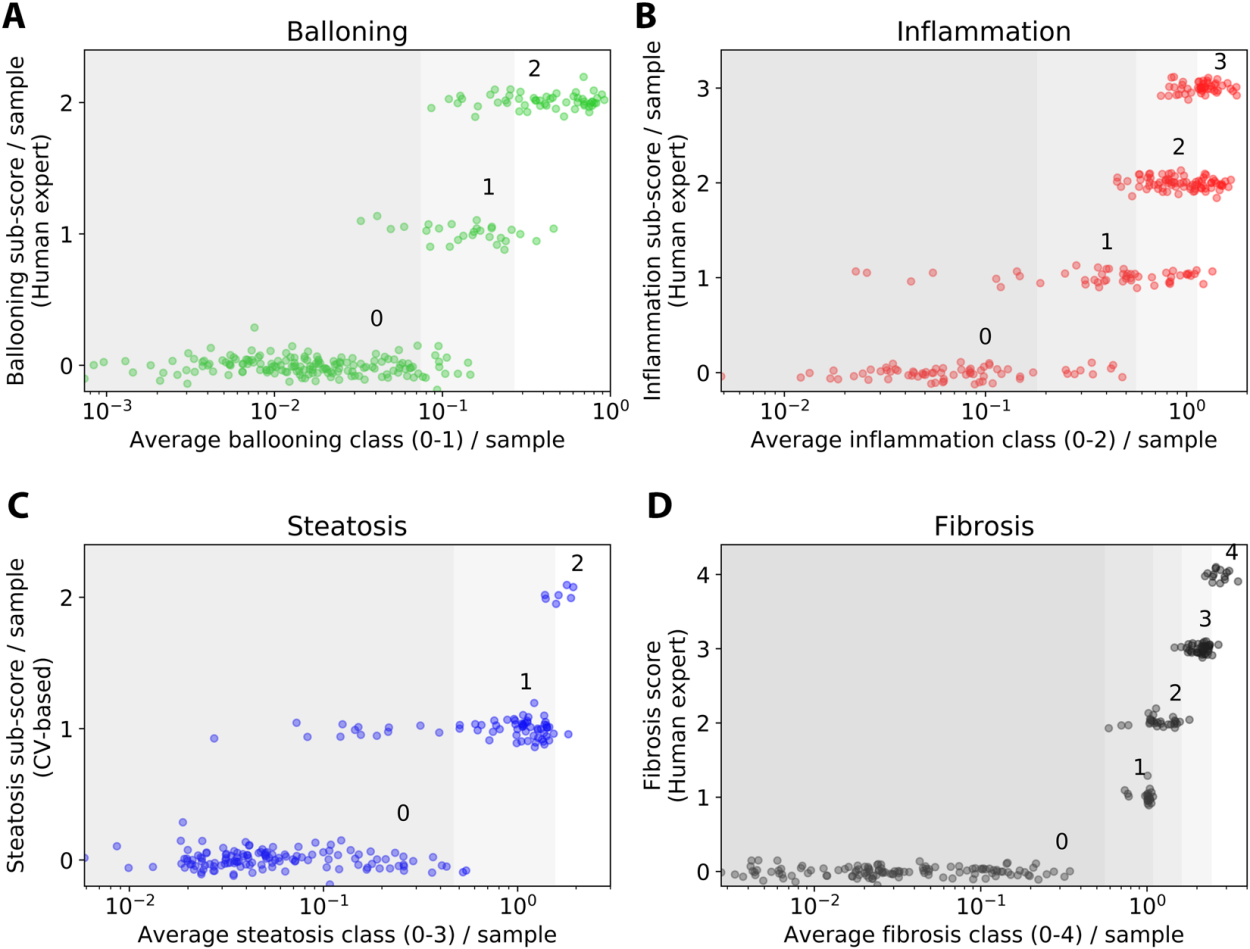
Deep learning-based average class per liver section (horizontal) compared to the pathologist’s score (vertical) on (**A**) ballooning, (**B**) inflammation, (**C**) steatosis, and (**D**) fibrosis. Dots are individual liver sections. Pathologist’s scores on the vertical axis have added jitter for visualization purposes. To obtain the discrete pathologist scores used in research and the clinic, thresholds are applied to the deep learning-based scores. Optimized thresholds were found using a Monte Carlo algorithm. Gray shaded areas show the resulting intervals.

Figure 5 shows a plot of averaged weighted class per liver section against the ground truth of over 200 experiments covering several years and various experimental conditions. Values on the horizontal axis show a wide distribution of states, even for a single pathologist score on the vertical axis. This highlights the much higher resolution of the continuous score compared to the values of the discrete ground truth. The correlation of the discrete scores and the continuous scores is indicated by the stair-like shape.

To map the continuous scores to the widely adopted discrete pathologist-like scores, we applied thresholds to divide the score into intervals. A Monte Carlo search algorithm was implemented to find optimal thresholds with minimized error of the deep learning-based scores compared to the ground truth scores. Thresholds were randomly varied in an iterative fashion to minimize the quadratic deviation of the deep learning-based score and the pathologist’s ground truth. Gray shaded areas in Fig. 5 show optimized thresholds after this procedure.

Table 2 shows an evaluation of the mapping performance on the dataset to determine the thresholds. The mapping performance was additionally tested on an independent held-back set (test data) to better assess the performance on new data.

**Table 2:** Mean average error (MAE) and Cohen’s κ of the mapped scores.

	MAE (best = 0)		Cohens κ (best = 1)	
	Train	Test	Train	Test
Ballooning	0.16	0.30	0.70	0.42
Inflammation	0.36	0.45	0.51	0.40
Steatosis	0.10	0.04	0.80	0.92
Fibrosis	0.08	0.14	0.88	0.81

MAE indicates the typical deviation from the ground truth in units of the pathologist’s score with 0 as the optimal value. Cohen’s κ indicates the agreement of the deep learning-based approach and the ground truth with 1 as perfect agreement and values of 0 or lower as random agreement.

To allow estimating the expected deviation in terms of units of the pathologist score, we computed the mean absolute error (MAE). In addition, we computed Cohen’s κ score to quantify the agreement of DL scores with the pathologist’s ground truth (with κ = 1 for perfect agreement and values of κ <= 0 for random agreement). Further evaluation metrics can be found in Table S1.

Fibrosis (MAE = 0.14, κ = 0.81 on the test data) and steatosis (MAE = 0.04, κ = 0.91 on the test data) was mapped with very good agreement and very low error. Ballooning (MAE = 0.30, κ = 0.42 on the test data) and inflammation (MAE = 0.45, κ = 0.40) resulted in more variability compared to the ground truth, but can still be mapped with moderate expected error (as shown by MAE). In cases of deviations, almost all of them were in an adjacent class, i.e. not totally off.

Since Cohen’s κ is known for the components of the Kleiner score for the agreement levels of different pathologists^18^, its computation allowed a direct comparison with the agreement of the deep learning-based algorithm with the pathologist providing the ground truth data. The previously reported κ values for different pathologists were κ = 0.84 (fibrosis), κ = 0.79 (steatosis), κ = 0.56 (ballooning), and κ = 0.45 (inflammation)^18^. Also in this previous study with different human annotators, ballooning and inflammation were the most challenging features in terms of inter-observer variability. We therefore assume that a higher intra-observer variability for ballooning and inflammation can also be expected, which affects the accuracy of the obtained thresholds. Finally, the slightly higher performance on the train set compared to the test set indicates some amount of over-adaptation on the training set.

In the future, two aspects will be most beneficial to further improve and generalize the models: first, additional training of the CNN models since CNNs get increasingly better with the amount of data due to their very high learning capacity^28^. Focusing on more challenging types of images will help to further optimize the classification performance on such cases (e.g. discriminating ballooning from glycogen storage combined with (micro-)-steatosis within one cell; which represents an example of a challenging case). Second, extending the training data used for the threshold determination. Here it will be of particular benefit to obtain more dense data distributions for all scores of the ground truth (pathologist scores) since this affects the obtained thresholds.

## Conclusion

The rapid advances in deep learning-based approaches for image recognition now allow the automation of tasks which, until recently, were the exclusive domain of human experts.

Here, we described a new approach to automate the Kleiner score for liver tissue sections of animal models for NAFLD/NASH. The approach is based on simple computational building blocks (classification CNNs and threshold fitting). The annotation effort for the pathologist is kept moderate as it relies on tile sorting instead of the annotation of complex regions.

In addition to the discrete pathologist-like scores, the approach provides continuous valued readouts on the features most relevant for NASH. Although the data shown here were developed for animal models, we assume that they can be adapted to human samples by training with sufficient amounts of human liver tissue.

In our opinion, the pathologist of the future will be supported increasingly by automated analysis systems as the one described here. Such analysis systems require an increasing collaboration of pathologists with computer scientists. This collaboration will be crucial for improved automation, standardization and the generation of novel readouts.

Even if built carefully, deep learning-based systems can be mistaken, and it is therefore important to incorporate result verifications into an analysis workflow. Errors are particularly likely if variations in morphology or histological staining appear, which are not sufficiently represented in the training data. In these cases, retraining on the tile-level and using additional annotated slides for threshold mapping are recommended. With increasing amounts of data, these systems should become more and more robust in a wide range of possible experimental conditions.

As a next step, experts could define further relevant histological features of pathological processes of the liver (e.g. different types of inflammation). The approach described here could serve as a starting point for quantifying such novel features. It is completely built from open-source building blocks and can therefore be easily extended.

These features might be added to the four features shown here and result in a multi-dimensional histological feature vector describing the pathological trajectory of the liver. In combination with the continuous readouts, this could lead to histopathological readouts with improved information content and therefore optimize the use of the experimental animal models. This is important in terms of the ethical use of animals and an improved drug discovery process.

## Methods

### Animals

Liver sections from previous animal studies (2014 and 2019) were reanalyzed. Mice (C57BL/6JRj) and rats (RjHan:WI) at different ages from Charles River (Sulzfeld, Germany), Taconic (Rensselaer, United States) or Janvier (Le Genest-Saint-Isle, France) were used. Animals included healthy controls and NAFLD/NASH models. The disease models comprised established approaches, such as CCl_4_ or CDAA^17,29,30^ and resulted in varying degrees of morphological changes correlated with NASH.

Animals were maintained in accordance with German national guidelines, legal regulations and the guidelines of the Association for Accreditation of Laboratory Animal Care. Experiments were performed after permission from the Regierungspräsidium Tübingen, Germany.

### Tissue samples and staining

Animals were sacrificed by using an overdose of pentobarbital. Livers were removed and the right liver lobe was fixed by incubating in para-formaldehyde and embedded in paraffin according to standard protocols. A 3 μm thin section of a central cut of the liver lobe was stained with Masson’s trichrome. Staining quality was routinely controlled by microscopy before further analysis. This reduced the chance of analyzing inaccurately stained samples, which might result in classification errors.

### Microscopy

Microscopic analysis of whole slides was performed with a Zeiss AxioScan Z1 scanner (Carl Zeiss, Jena, Germany) with a 20x objective in bright field illumination with a pixel resolution of 0.22 µm/px. Images were exported to BigTIFF with a factor of 1:2 at 0.44 µm/px using the software Zen Blue 2.6.(Carl Zeiss, Jena, Germany; https://www.zeiss.com/microscopy/int/products/microscope-software/zen.html). Also images from conventional bright field microscopes can be used, if acquired at 20x with the pixel resolution defined above.

### Manual scoring procedure

Liver sections were assigned to discrete scores quantifying the histopathological features of ballooning (0–2), inflammation (0–3), steatosis (0–3) and fibrosis (0–4) following an established scoring system^18^. The scoring was performed by an experienced veterinary pathologist (B.S.) in a blinded setting (randomized slides without knowledge of experimental group).

### Tile generation

Image tiles in two dimensions were generated from exported BigTIFFs by evenly covering the image with non-overlapping adjacent tiles: Low magnification tiles were generated from an area of 897 × 897 px² and exported with a downscaling factor of 1:3 to 299 × 299 px² at 1.32 µm/px. High magnification tiles were generated from an area of 299 × 299 px² and exported without further downscaling at 0.44 µm/px. Low magnification tiles were used for the fibrosis model and high magnification tiles for the ballooning, inflammation and steatosis models. Halcon image processing software (MVTec Software GmbH, Munich, Germany) was used for tile generation. The final tile size of 299 × 299 px² was selected to directly match the input dimension of the CNN backbone used (Inception-V3). The pixel resolutions of 1.32 µm/px (fibrosis tiles) and 0.44 µm/px (ballooning, inflammation and steatosis tiles) allowed the pathologist to sufficiently identify the relevant features within each tile.

### Deep learning

For each histological feature (fibrosis, ballooning, inflammation, and steatosis), a distinct CNN model was trained. The Inception-V3^31^ CNN architecture was used in the Keras^32^ implementation by using pre-trained weights from training on ImageNet^33^ to utilize pre-build convolutional filters. These filters were subsequently fine-tuned by training with the respective dataset of histological images (fibrosis, ballooning, inflammation, and steatosis). The original fully-connected layers after the last convolution were discarded and replaced by global average pooling, followed by a dropout layer^34^ with a dropout rate of 0.5 to reduce overfitting and a fully connected layer with outputs corresponding to the number of classes in each model (fibrosis: 6, ballooning: 3, inflammation: 4, steatosis: 5). Outputs were normalized to 1 using the softmax function.

The CNNs were trained with stochastic gradient descent with an initial learning rate of η = 0.5·10^−4^ and a momentum of μ = 0.9 to minimize the categorical cross-entropy loss on the validation data. If the loss on the validation data did not decrease for more than two epochs, the learning rate was reduced by multiplying with a factor of 0.2 to a minimal learning rate of η = 10^−7^. No further hyperparameter tuning was performed since previous experience with these parameters and Inception-V3 resulted in very good recognition performances with higher agreement levels in tile recognition compared to two human experts^25^. Class imbalances were equalized by oversampling. All layers were kept trainable. During training, the images were augmented by random rotations *θ* in the interval $\theta \,\epsilon \,[-\frac{\pi }{4},\frac{\pi }{4}]$, horizontal and vertical flips and shifts *d* in width and height in the interval $d\,\epsilon \,[-30,30]$ px.

### Training and validation tile data for deep learning

To train the CNN classifiers for fibrosis, ballooning, inflammation, and steatosis tiles were annotated by an experienced veterinarian. Rat and mouse samples were combined due to their highly comparable morphology.

For fibrosis, tiles were sorted into classes 0, 1, 2, 3, 4 and ignore with labels corresponding to the macroscopic fibrosis score as defined by Kleiner *et al*.^18^.

For ballooning, classes 0, 1 and ignore were defined as follows: 0 corresponds to a tile without a ballooning cell, and 1 corresponds to a tile with one or more ballooning cells. This differs from the macroscopic sub-score (per liver section) with a range of 0, 1, 2 since macroscopically, the ballooning sub-scores are defined by “none” (0), “few” (1), or “many” (2) ballooning cells^18^, which cannot be reflected in the small dimensions of a tile.

For inflammation, tiles were sorted into classes 0, 1, 2 and ignore. 0 (negative or regarded as background) corresponds to no inflammatory cells or cell cluster of less than three inflammatory cells or less than 5 disseminated inflammatory cells visible on a tile, 1 to cell cluster between three and five inflammatory cells and/or between five and ten disseminated inflammatory cells per tile, and 2 to cluster of more than 5 inflammatory cells and/or more than ten disseminated inflammatory cells per tile. Also, in this case, a different definition had to be used on the tile level compared to the macroscopic inflammation sub-score per liver section.

For steatosis, tiles were automatically sorted into classes of 0, 1, 2, 3 and ignore according to the area covered by steatosis per tile (0: <5%; 1: >=5% <33%; 2: >=33% <66%; 3 >= 66%) as determined via a classical computer vision approach using Halcon image processing software. Briefly, the area fraction of bright areas within a predefined roundness and size range were detected. Subsequently pre-sorted tiles were manually curated. This approach was used for this sub-score since, in our experience, computer-vision based area quantifications are more accurate than human assessments.

The ignore class was used in all cases to allow the CNNs to sort tiles out where insufficient information was shown (e.g. border with more than 50% empty space, out of focus, mostly blood, or staining artifacts). Around 90% of the data were used for training of CNNs and 10% was randomly selected for validation to test the classifier’s performance.

### Class activation maps

Class activation maps were computed according to the method described by Zhou *et al*.^26^ in an adaptation for Inception V3. Briefly, the last convolutional layer contains spatial information, which can be used for visualization. For Inception-V3, the output of the last convolutional layer (*f*_*kxy*_) is a tensor of dimension 2048 × 8 × 8, with the last two dimensions *x*, *y* in spatial direction and the first dimension *k* as feature maps (i.e. filtered image properties). The next layer, global average pooling, combines the features spatially, i.e. ${F}_{k}=\sum _{x,y}\,{f}_{kxy}$. Subsequently, the output *s*_*c*_ for class *c* is computed by a fully connected layer with weights *w*_*ck*_ (note: bias term is omitted as in Zhou *et al*.^26^) which is: ${s}_{c}=\sum _{k}\,{w}_{ck}\sum _{x,y}\,{f}_{kxy}$, or ${s}_{c}=\sum _{x,y}\,\sum _{k}\,{w}_{ck}\,{f}_{kxy}=\sum _{x,y}\,{M}_{c}(x,y)$. Therefore, the term ${M}_{c}(x,y)=\sum _{k}\,{w}_{ck}\,{f}_{kxy}$ contains spatial information which can be scaled to the input image to visualize the most activated areas for a decision for class *c*.

### Processing of classification results and aggregation per liver

The final softmax output of the CNN can be interpreted as a confidence vector (*q*_0_ … *q*_*c*−1_ *q*_*c*_)^*T*^ for the classes of each model with ‘ignore’ as class *c*.

Only slides in which ignore was not the predicted class were considered further (i.e. *q*_*c*_ ≠ *arg max*(*q*_0_ … *q*_*c*−1_ *q*_*c*_)^*T*^). To correct for cases where a tile contained an amount of “ignore” (e.g. some amount of edge was present, but also a ballooning cell), we re-normalized the confidences to a sum of 1 without “ignore” as follows:

$${p}_{j}=\frac{{q}_{j}}{({\sum }_{i=0}^{C-1}\,{q}_{i})}$$

The normalized confidences *p*_*j*_ were summarized as the weighted average class score 〈*ip*_*i*_〉:

$$\langle i{p}_{i}\rangle =\mathop{\sum }\limits_{i=0}^{C-1}\,i{p}_{i}$$

To aggregate the entire liver sample, the average class score *x* of the *N* tiles of a liver sample was calculated:

$$x=\frac{1}{N}\sum _{liver}\,\mathop{\sum }\limits_{i=0}^{C-1}\,i{p}_{i}$$

This continuous readout can be used directly or mapped to a pathologist score.

### Mapping of aggregated scores to pathologist scores

The average class score *x* for liver samples was compared to the pathologist’s Kleiner score (ground truth) for 258 cases from 2014–2019 (Fig. 5). The mapping of this continuous score *x* to a discrete pathologist-like score *a* ∈ *A*{0, 1, …} was done by using a set of thresholds *t*_*i*_. For example, in a case of |*A*| = 3 discrete pathologist scores (i.e. ballooning with scores *A* = {0, 1, 2}), the mapping function *s*(*x*) is defined by:

$$s(x)=\{\begin{array}{ll}0, & 0\le x < {t}_{0}\\ 1, & {t}_{0}\le x < {t}_{1}\\ 2, & {t}_{1}\le x < {t}_{2}\end{array}$$

Other mapping functions were defined accordingly.

A Monte Carlo search algorithm was implemented to find a set of optimal thresholds which minimized the quadratic error *E* of scores using the current mapping function *s*_*k*_(*x*) compared to the ground truth ${g}_{k}\,\epsilon \,A$ (pathologists’ scores) over all *k* = 1 … *K* liver samples used to fit the optimal thresholds.

$$E=\sum _{a\,\in \,A}\,{f}_{a}\sum _{{g}_{k}=a}\,{({s}_{k}(x)-{g}_{k})}^{2}$$

The left sum is calculated over all unique scores in *A*. The right sum is calculated over all examples and measures the quadratic deviation of the mapping function *s*_*k*_(*x*) and the ground truth *g*_*k*_. Weighting factors *f*_*a*_ for each *a* ∈ *A* = {0, 1, …} were used to compensate for imbalance of pathologist scores and are defined as.

$${f}_{a}=\frac{K}{{k}_{a}}$$

This compares the total number of examples *K* with the number of examples *k*_*a*_ with a ground truth score of *a*, i.e. *k*_*a*_ = |*g*_*k*_ = *a*|.

Thresholds *t*_*i*_ were initialized within the possible value range of all *x* such that *t*_*i*−1_ < *t*_*i*_. Empirically, an initialization of the thresholds *t*_*a*_ to the 75^th^ quantiles of the set of {*x*} corresponding to a ground truth value (pathologist score) of *a* resulted in fast convergence.

Whenever a new best set of thresholds was found, this set was used as new starting set and new thresholds were created by adding Gaussian distributed random numbers to the previous best set of thresholds (standard deviation *σ* = 0.15). If the error *E* using the new set was lower, this was used as a new best set of thresholds *t*_*i*_. This procedure was repeated until convergence of the error.

An additional dataset of 92 scored livers was not used to determine the threshold (or for the per-tile training), but rather used as held-back test set. Score mapping performance was evaluated with mean absolute error, weighted F1 score, weighted precision, weighted recall, accuracy and Cohen’s κ score using scikit-learn^35^.

## Supplementary information

Supplemental information

## Data Availability

All relevant data required for training and validation of the four CNN models and for the analysis of new liver samples can be found at Open Science Framework (https://osf.io/p48rd/). Python scripts to generate the data presented in the manuscript are accessible via github: (https://github.com/FabianHeinemann/Deep_learning_for_liver_NAS_and_fibrosis_scoring).
