# Supplementary material for: Deep learning enables pathologist-like scoring of NASH models
Source: Sci Rep. 2019 Dec 5;9:18454. doi: 10.1038/s41598-019-54904-6 (PMC6895116; doi:10.1038/s41598-019-54904-6)
Supplement: Supplementary file 1 — Supplemental information [file 41598_2019_54904_MOESM1_ESM.pdf]

Supporting material:

Deep learning enables pathologist-like scoring of NASH models

Fabian Heinemann[1,*], Gerald Birk[1], Birgit Stierstorfer[1]

[1] Target Discovery Science, Boehringer Ingelheim Pharma GmbH & Co. KG, 88397 Biberach an der Riß, Germany

[*] Corresponding author: fabian.heinemann@boehringer-ingelheim.com

	**MAE** (best = 0)		**F1** (best = 1)		**P** (best = 1)		**R** (best = 1)		**A (%)** (best = 100%)		**Cohen's κ** (best = 1)	
	**Train**	**Test**	**Train**	**Test**	**Train**	**Test**	**Train**	**Test**	**Train**	**Test**	**Train**	**Test**
**Ballooning**	0.16	0.30	0.86	0.72	0.90	0.77	0.84	0.72	84.2	71.7	0.70	0.42
**Inflammation**	0.36	0.45	0.63	0.59	0.64	0.64	0.63	0.57	64.0	56.5	0.51	0.40
**Steatosis**	0.10	0.04	0.90	0.96	0.90	0.96	0.90	0.96	90.3	95.7	0.80	0.91
**Fibrosis**	0.08	0.14	0.92	0.85	0.93	0.89	0.92	0.86	92.4	85.9	0.88	0.81

Table S1: Error metrics of the mapping process. Mean average error (MAE). Weighted F1 score (F1) (geometric mean of precision and recall), weighted precision (P), weighted recall (R), accuracy of exact matches (A) and Cohen's κ. Metrics were computed on the dataset to determine the thresholds (training set) and on an independent data set (test set).

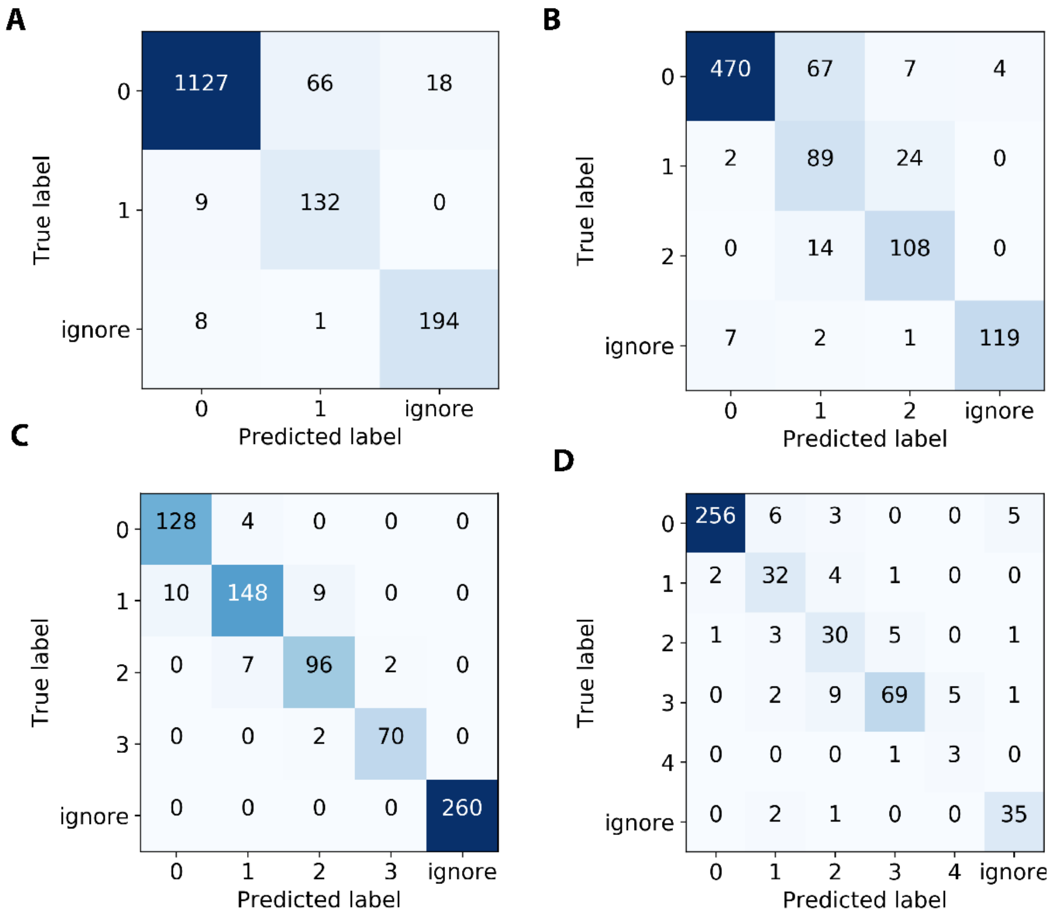

Figure S1: Confusion matrices of the four convolutional neural networks (CNN) on A) ballooning, B) inflammation, C) steatosis, and D) fibrosis. Classification was done on held back validation data. The result of the CNN classification is shown in columns compared to the ground truth provided by the expert pathologist in rows. Numbers are numbers of tiles. In most cases, values are found on the diagonal, corresponding to exact agreement. In the majority of cases, disagreement is found on neighboring fields, presumably caused by inherent ambiguity (e.g. transition cases of the biological images between the discrete classes) of parts of the images and by images which are inherently challenging to classify (e.g. insufficient information on a tile for confident classification by pathologist and / or CNN).
